# Mediating Roles of PPARs in the Effects of Environmental Chemicals on Sex Steroids

**DOI:** 10.1155/2017/3203161

**Published:** 2017-07-27

**Authors:** Qiansheng Huang, Qionghua Chen

**Affiliations:** ^1^Key Lab of Urban Environment and Health, Institute of Urban Environment, Chinese Academy of Sciences, Xiamen 361021, China; ^2^Center for Excellence in Regional Atmospheric Environment, Institute of Urban Environment, Chinese Academy of Sciences, Xiamen 361021, China; ^3^The First Affiliated Hospital of Xiamen University, Xiamen 361003, China

## Abstract

Peroxisome proliferator-activated receptors (PPARs) are ligand-activated nuclear receptors that are widely involved in various physiological functions. They are widely expressed through the reproductive system. Their roles in the metabolism and function of sex steroids and thus the etiology of reproductive disorders receive great concern. Various kinds of exogenous chemicals, especially environmental pollutants, exert their adverse impact on the reproductive system through disturbing the PPAR signaling pathway. Chemicals could bind to PPARs and modulate the transcription of downstream genes containing PPRE (peroxisome proliferator response element). This will lead to altered expression of genes related to metabolism of sex steroids and thus the abnormal physiological function of sex steroids. In this review, various kinds of environmental ligands are summarized and discussed. Their interactions with three types of PPARs are classified by various data from transcript profiles, PPRE reporter in cell line, in silico docking, and gene silencing. The review will contribute to the understanding of the roles of PPARs in the reproductive toxicology of environmental chemicals.

## 1. Introduction

Peroxisome proliferator-activated receptors (PPARs) are ligand-activated nuclear receptors which are widely involved in various physiological and pathological processes [1]. The family contains three subtypes (PPAR*α*, PPAR*β*/*δ*, and PPAR*γ*) with various ligand specificity, tissue distribution, and biological function. PPARs are detectable in various compartments of the reproductive system, including hypothalamus, pituitary, testis, ovary, uterus, and adrenal and mammary gland. PPARs are widely involved in reproductive function, such as ovarian function, gestation, and communication between mother and fetus [2–4]. Sex steroids, also named as gonadal steroids, are defined as steroid hormones that interact with receptors of androgen, estrogen, and progesterone in vertebrates [5]. Sex steroids are produced by gonads (ovaries or testes) and adrenal glands. Further conversion could occur in other tissues such as livers and fats. PPARs are critical for the metabolism and physiological function of sex hormones [2, 6].

Large amounts of pollutants have been released into the environmental media as a consequence of rapid industrialization and urbanization. Exposure to pollutants has been reported to be a big risk for reproductive health [7]. The mechanism through which pollutants elicit adverse effects is still not fully understood. However, it is widely accepted that pollutants could adversely affect the reproductive function through disturbing the metabolism and function of sex steroids [8]. Pollutants could bind to PPARs and then modulate the PPAR signaling pathways involved in the reproductive function. Hydrophobic interactions are the primary driving force for the binding between pollutants and PPARs. Most of the amino acid residues are hydrophobic around the binding pocket which located inside the protein structure of PPARs [9]. The sequences of amino acids which form the pocket are conserved across species. Results from reporter cell lines also show that environmental ligands (BPA derivatives, phthalates, and PFAAs) share similar affinity for PPAR*γ* of zebrafish and human [10].

In this review, the interactions between PPARs and sex steroids are presented. Various kinds of PPAR ligands, especially environmental chemicals, are summarized. The pathways through which exogenous chemicals exert their impact on the metabolism and function of sex steroids via PPARs are depicted.

## 2. Interaction between PPARs and Sex Steroids

Androgens and estrogens are the primary types of sex steroids. Exogenous testosterone significantly inhibited the expression of PPAR*γ* in primary hepatocytes isolated from brown trout [11]. 17*β*-Estradiol could regulate the expression of PPAR*γ* in human peripheral blood eosinophils [12]. Additionally, precursors of sex steroids also interact with PPARs. For example, dehydroepiandrosterone (DHEA) induced elevated expression of both PPAR*α* and PPAR*β*/*δ* in the muscle of mice [13]. Conversely, PPARs have an important impact on sex steroids. Single nucleotide polymorphism (SNP) of PPARs significantly affected the level of sex steroids and was linked to hormone related diseases. For example, the SNP of PPAR*γ* at P12A (Pro12Ala, rs1801282) was linked to a gynecological disease: polycystic ovary syndrome (PCOS) as PCOS patients with CG genotype showed lower free testosterone and other hormones than that of GG genotype [14]. Peroxisome proliferators (PPs) are a group of chemicals which function through PPARs. PPs could impair the function of endocrine tissues by regulating the expression of phase I and phase II steroid metabolism enzymes [15], including P450 enzymes and 17*β*-hydroxysteroid dehydrogenase IV [16]. Apart from their impact on metabolism, PPs could also disturb the physiological function of sex steroids. They have been reported to mimic or interfere with the action of sex steroids and then induce reproductive disorders [17]. In addition, receptors of sex steroids were also reported to interplay with PPARs. For example, estrogen receptor alpha (ER*α*) binds to the PPRE sequence of PPAR*γ* and represses its transactivation in MCF-7 cells [18]. Bidirectional interplay occurs between PPAR*γ* and ER [19].

Sources of PPs contain endogenous and exogenous chemicals. Endogenous essential fatty acids (FAs) and their derivative eicosanoids are able to activate the PPAR signaling pathway [20]. 17*β*-Estradiol could suppress the expression of PPAR*α* regulating genes [21]. In addition to these endogenous chemicals, chemicals from environmental media, drugs, and other external sources are also reported to disturb the PPAR signaling pathway and then affect metabolism and function of sex steroids.

## 3. Environmental Chemicals as Exogenous Ligands

A lot of environmental chemicals act as exogenous ligands to PPARs. These chemicals are widely detectable in the human body and have received widespread public health attention [52]. PPARs have been regarded as a bridge to link the environmental chemicals and their health impact [53]. Chemicals which could modulate the PPAR signaling pathway and affect the sex steroids are classified and listed as follows. They are also shown in Table 1.

### 3.1. Phthalates

Phthalates were widely reported as reproductive toxicants. Fetal exposure to environmentally relevant di(2-ethylhexyl) phthalate (DEHP) decreased serum levels of steroid hormones in adult male mice and antagonism of PPAR*γ* diminished the toxic effect [22]. In our study, PPAR*γ* was thought to transduce the toxicity of DEHP at 0.2–2 *μ*M in both primary cultured endometrial cells and endometrial adenocarcinoma cell line (ishikawa) [23]. We also obtained consistent results in a marine fish model where the expressions of PPAR*γ* and aromatase were both enhanced after fish embryo exposure to DEHP at 0.1–1 mg/L [24]. In vivo, DEHP is metabolized to mono(2-ethylhexyl) phthalate (MEHP) which could activate both PPAR*α* and PPAR*γ* and then suppress the transcription of aromatase and estradiol production in the ovary. These have been verified both in rat ovarian granulosa cell models [26] and in the ovary of rat models [25]. Direct exposure to MEHP at the dose of 50 *μ*M also inhibited the expression of aromatase by activating PPAR*α* or PPAR*γ* in rat granulosa cells [27]. Due to the adverse health outcome of commonly used compounds, phthalate-alternative compounds have been emerging. Some of these chemicals also showed various affinities to PPARs and different influences on reproductive function according to docking studies [30, 54–56]. For example, diisononyl phthalate (DINP) showed DEHP-like affinity to PPAR*α*. Di(2-ethylhexyl) terephthalate (DEHT) has the paraisomer structure of DEHP and shows a very weak affinity to PPAR*α*.

### 3.2. Perfluoroalkyl Acids

Perfluoroalkyl acids (PFAAs), characteristic of fully fluorinated carbon chains, are widely used in consumer goods and industrial products. Concerns have arisen regarding human exposure and adverse outcome, especially due to extremely long biologic retention time [57]. Treatments with perfluorononanoic acid (PFNA), perfluorooctanoic acid (PFOA), perfluorodecanoic acid (PFDA), perfluoroundecanoic acid (PFUnDA), and perfluorooctane sulfonate (PFOS) all dose-dependently activated PPAR*α* using a PPRE reporter system [31, 33]. PFOS and PFOA are top two members in toxicological studies. Our study showed that the effects of PFOS (1–16 mg/L) were different on the expression of these three types of PPARs in the larvae of* O*.* melastigma* [37]. PFOA (5 mg/kg) exposure affected the expression of PPARs in a tissue dependent manner in fetal and postnatal CD-1 mice [34]. Both PFOS and PFOA can induce PPARs-mediated transcriptional activity determined by PPRE reporter assay [31, 33, 35, 36, 38, 39]. This led to alterations in immune response and other physiological processes [58]. However, the mediating roles of PPARs are not consistently recognized. Several studies confirm that PFOA can exert its toxicity independently of PPAR*α* [59, 60]. It is worth mentioning that four weeks' PFOA treatment (5 mg/kg) increased the expression of enzymes catalyzing the biosynthesis of steroid hormone and enhanced serum levels of progesterone in PPAR*α* knockout female mice [61]. Apart from the toxicological studies, a lot of epidemiological studies also revealed the effects of PFOA on reproduction. Positive or negative association was reported by publications from C8 science panel [62–64]. Different members of PFAAs show various impacts on PPAR signaling due to their various chain lengths and functional groups [65, 66]. Thus, toxicities of other family members are being studied. For example, perfluorododecanoic acid (PFDoA) administration (3 mg/kg) led to reduced serum levels of 17*β*-estradiol in prepubertal female rats; however, the roles of PPARs have not been verified yet [67].

### 3.3. Bisphenol A (BPA) and Its Derivatives

BPA is widely used in plastic bottles, paper, and other daily commodities. Due to structural similarity with 17*β*-estradiol, the estrogenic activity of BPA via ER activation was widely studied. In addition to ERs, BPA also show an affinity to human PPAR*γ* as confirmed by data from docking and PPRE reporter studies [9, 42, 43]. The affinity was ranked as ERR*γ* > ER*α* > PPAR*γ*. BPA exposure (0–100 *μ*M) led to reduced expression of aromatase and decreased level of E2 secretion in human ovarian granulosa cells, which also happened after overexpression of the PPAR*γ* [44]. By contrast, the same level range of BPA showed no significant effect on the expression of both PPAR*γ* and aromatase in human endometrial stromal fibroblast cells [68]. To be noted, low dose effects were observed in the toxicology of BPA. A low dose was considered to be a dose below the range typically used in toxicological studies of chemicals [69]. Biphasic U- or inverted U-shaped dose-response curves have been observed when evaluating the effects of BPA on reproduction and other health outcomes. Competitive binding to PPARs and other receptors between BPA and sex hormone might contribute to this low dose effect [70]. Derivatives of BPA could also interfere with PPARs. Brominated or chlorinated derivatives of BPA display their adverse impact through PPARs. Both tetrabromobisphenol A (TBBPA) and tetrachlorobisphenol A (TCBPA) show binding affinity to PPAR*γ* as indicated by reporter cell lines [40, 71]. Of further note, TBBPA (0.01–10 *μ*M) can also induce the expression of aromatase and thus enhance estrogen synthesis independently of PPAR*γ* in human choriocarcinoma JEG-3 cells [72, 73].

### 3.4. Dioxin-Like Chemicals

Dioxin and its structure-like chemicals are widely accepted as the ligands to aryl hydrocarbon receptor (AHR). They also interplay with PPARs as confirmed by in silico docking experiments [47]. An in vivo study using male rat model revealed that polychlorinated biphenyls 126 (PCB126) exposure at the dose of 5 *μ*mol/kg inhibited the mRNA expression of PPAR*α* and its downstream genes acyl-CoA oxidase (Acox1) and hydroxy-3-methylglutaryl-CoA synthase 2 (Hmgcs2) in liver [48]. In our study using both cells and mice models, PCB126 exposure at human relevant levels induces the expression of HSD17B7 and enhances the secretion of estradiol in endometrium [7]. Molecular evidence confirmed the existence of two PPRE sites at the promoter of cytochrome P4501A1 (CYP1A1). Thus, direct activation of CYP1A1 by PPAR*α* without AHR might be a new pathway to link PCBs and PPARs [74, 75]. However, strong evidence is still needed to confirm the link.

### 3.5. Pesticides

A lot of pesticides show disrupting effects on metabolism and function of sex steroids, such as deltamethrin [76], linuron [77], and methomyl [78]. The mediating function of PPARs is being studied in the toxicity of pesticides. For example, a large in vitro reporter gene assay screening study with 200 pesticides showed that PPARs did not have a major role in the toxicity of pesticides. Various kinds of pesticides were examined including 29 organochlorines, 11 diphenyl ethers, 56 organophosphorus pesticides, 12 pyrethroids, 22 carbamates, 11 acid amides, 7 triazines, 8 ureas, and 44 others. Results showed that only three (diclofop-methyl, pyrethrins, and imazalil) could activate PPAR*α* and none of them could activate PPAR*γ*. The agonist roles of these three pesticides were further confirmed in mice [79]. In a study that has raised wide ecotoxicological concern, the mRNA level of PPAR*γ* was induced by DDT at the doses of 100 pM-10 *μ*M in human mesenchymal stem cells [49]. To be noted, direct evidence is still expected on the effects of pesticides on sex steroids through PPAR signaling.

### 3.6. Other Pollutants

Organotin compounds are ubiquitously present in environment media. The compounds have been reported to alter endocrine functions in juvenile salmon and human choriocarcinoma cell lines [50, 80]. Tributyltin (TBT) could activate all three types of RXR (retinoid X receptor): PPAR*α*, PPAR*β*/*δ*, and PPAR*γ* heterodimers by PPRE luciferase experiment [51]. 2,4-Dichlorophenoxyacetic acid (2,4-D) is a possible endocrine disruptor. Treatment with 2,4-D decreased the level of testosterone in mice serum and testis through inhibiting the expression of 3-hydroxy-3-methylglutaryl coenzyme A synthase 1 and reductase, which led to decreased cholesterol levels. PPAR*α* exerted a critical role as its silencing diminished these toxic effects [6].

### 3.7. Pollutant Mixtures

In addition to individual pollutants, chemical mixtures also display reproductive toxicity through PPARs. Combined exposure to 2,3,7,8-tetrachlorodibenzo-p-dioxin (TCDD) and DEHP led to decreased estradiol synthesis in human granulosa cell line—KGN. Direct activation of AHR and transactivation of PPARs are indispensable parts in this molecular response pathway [81]. Chemical mixtures extracted from natural water could also disturb the function of steroid hormones where PPARs act as key regulators [82, 83]. In our study, cotreatment with DEHP and PCBs promoted the expression of PPAR*γ* but not the other PPAR types in mice liver [84].

## 4. Conclusion and Perspectives

PPARs, especially the subtype of *α* and *γ*, have important roles in mediating the toxicological outcomes caused by environmental ligands. Various kinds of environmental pollutants show impacts on the metabolism and function of sex steroids through disturbing the PPARs signaling pathways. The interactions between PPARs and environmental chemicals have been revealed through various approaches including molecular docking, PPRE reporter, transcript profiles, and gene silencing which are performed in silico, in vitro, and in vivo. Future studies that should be carried out include (1) structural biological studies on crystal structures of pollutants bound to PPARs and (2) further evaluation of the crosstalk between PPARs and other classical nuclear receptors, such as ER and AHR. These studies will help reveal the roles of PPARs in the toxicology of environmental pollutants on sex steroids.

## Figures and Tables

**Table 1:** Various kinds of environmental activators of PPARs and their impact on sex steroids.

Chemicals	PPARs subtype	Methods	Effects on sex steroids	Experimental model	References
DEHP	*α*, *γ*	Docking, transcript profiles, antagonism	Enhanced expression of aromatase, altered levels of estradiol	Rat, endometrial cells, fish	[22–25]
MEHP	*α*, *β*/*δ*, *γ*	Antagonism, transcript profiles	Decreased expression of aromatase and estradiol production	Rat ovarian granulosa cells, rat model, human liposarcoma cells, 3T3-L1 cells	[25–29]
DEHT	*α*	Docking	No significant impact	Rat model	[30]
PFNA	*α*, *β*/*δ*, *γ*	Transcript profiles, PPRE reporter	Elevated expression of CYP4A	Zebrafish, monkey kidney CV-1 cell line	[31, 32]
PFOA	*α*, *γ*	PPRE reporter, transcript profiles, antagonism, gene silencing	/	Monkey kidney CV-1 cell line, mice	[31, 33–36]
PFOS	*α*, *γ*	PPRE reporter transcript profiles, antagonism, gene silencing	/	Monkey kidney CV-1 cell line, *O. melastigma*, mice	[31, 33, 35–39]
PFDA	*α*	PPRE reporter	/	Monkey kidney CV-1 cell line	[31]
PFUnDA	*α*	PPRE reporter	/	monkey kidney CV-1 cell line	[31]
TBBPA	*γ*	PPRE reporter	Increased apelin expression and secretion	Epithelial ovarian cancer cell line (OVCAR-3)	[40, 41]
TCBPA	*γ*	PPRE reporter	Increased apelin expression and secretion	OVCAR-3	[40, 41]
BPA	a, *β*/*δ*, *γ*	Transcript profiles, PPRE reporter, docking	Decreased expression of aromatase and estradiol production	Human ovarian granulosa cell, mouse embryo fibroblasts, OVCAR-3	[41–46]
PCB77	a, *β*/*δ*, *γ*	Docking, antagonism	/	/	[47]
PCB118	a, *β*/*δ*, *γ*	Docking, antagonism	/	Cell model, mice model	[7, 47]
PCB126	*α*	Transcript profiles	Altered the secretion of estradiol	Rat model, mice model	[48]
DDT	*γ*	Transcript profiles	Enhanced expression of CYP4A	Human mesenchymal stem cells	[49]
2,4-D	*γ*	Transcript profiles, gene silencing	Decreased cholesterol levels	Mice leydig cells, mice model	[6]
TBT	a, *β*/*δ*, *γ*	PPRE reporter	Inhibition of gonad development	Juvenile salmon, cell model	[50, 51]
EE2	*γ*	Transcript profiles		Brown trout	[11]

“/” indicated data is not available as we known; The experimental methods to support the interaction between chemicals and PPARs are depicted as follows, *Antagonism*: PPARs was antagonized by specific antagonist. Then, the effects elicited by chemicals were re-assessed. If the effects were diminished or enhanced, the mediating roles of PPARs could be confirmed; *Transcript profiles*: Transcript profiles of PPARs were modulated by chemicals treatment; *Docking*: Computing methods to predict the structural binding between chemicals and PPARs; *PPRE reporter*: Reporter system was constructed by transfecting the luciferase reporting plasmid containing PPRE sequence into the cells. Then, the cells were treated with chemicals to determine whether chemicals functioned through activating PPARs; *Gene silencing*: The expression of PPARs was inhibited by RNAi or gene knockout. Then, the effects elicited by chemicals were re-assessed. If the effects were diminished or enhanced, the mediating roles of PPARs could be confirmed; *Abbreviation*: di-(2-ethylhexyl) phthalate, DEHP; mono-(2-ethylhexyl) phthalate, MEHP; di(2-ethylhexyl) terephthalate, DEHT; perfluorododecanoic acid, PFDoA; perfluorononanoic acid, PFNA; perfluorooctanoic acid, PFOA; perfluorodecanoic acid, PFDA; perfluoroundecanoic acid, PFUnDA; perfluorooctane sulfonate, PFOS; BPA diglycidyl ether, BADGE; tetrabromobisphenol A, TBBPA; tetrachlorobisphenol A, TCBPA; bisphenol A, BPA; polychlorinated biphenyls, PCB; dichlorodiphenyltrichloroethane, DDT; 2,3,7,8-tetrachlorodibenzo-p-dioxin, TCDD; 2,4-dichlorophenoxyacetic acid, 2,4-D; organotin, TBT; ethinylestradiol, EE2.
